# Practical and methodological challenges in the design and implementation of a cluster-randomised feasibility trial of the management of urinary incontinence after stroke

**DOI:** 10.1186/1745-6215-12-S1-A151

**Published:** 2011-12-13

**Authors:** Chris J Sutton, Lois Thomas, Denise Forshaw, Caroline L Watkins

**Affiliations:** 1 School of Health, University of Central Lancashire, Preston, Lancashire, PR1 2HE, UK

## Objective

To evaluate design and implementation issues in an NIHR-funded feasibility trial of the management of urinary incontinence after stroke in a secondary care setting.

## Methods

Twelve stroke services were cluster-randomised to 3 intervention groups (systematic voiding programme with/without supported implementation; usual care) in 4 strata based on: having separate/combined acute and rehabilitation units; above/below median performance on the ‘nine key indicators of stroke care’ in the National Sentinel Stroke Audit (NSSA) [1]; number of annual stroke admissions. Target recruitment was 780 patients overall; the recruitment period was 9 or 12 months, depending on a Trust’s annual stroke admissions, to reduce variability in numbers across services. Each service gained an additional 2.8 whole time equivalent health care assistants (HCAs) supporting introduction of the intervention or maintaining parity of staffing in ‘usual care’ services.

## Results

Recruiting services proved problematic, with excess treatment costs and service support cost being unavailable in some regions locally. The Comprehensive Local Research Network helped negotiate service support and excess treatment costs in England, leading to the recruitment of 8 sites. We were encouraged in recruitment of Welsh services, although differences in funding structure and governance processes caused further delays. The Welsh Assembly’s commitment to stroke services meant that funding from them was accessible, although acquiring Health Board funding was more problematic. R&D approval was slow in all 12 Trusts, in some taking almost a year. Appointment of staff was often delayed by current vacancy control procedures in the NHS.

All English services are now recruiting, with larger ones recruiting satisfactorily. Smaller services typically started later and have had greater difficulty meeting their lower targets. Further recruitment strategies introduced recently include a newsletter about trial progress. Welsh sites are commencing recruitment in late summer/autumn 2011. The unplanned staggered start of sites caused a loss of allocation concealment, although there is no evidence that this impacted on site participation. A 9-month trial extension has been approved, with participant recruitment continuing until July 2012.

## Conclusions

Designing and implementing a cluster-randomised trial of a complex intervention can be difficult, particularly so in the current financial climate in the NHS. This will impact on the feasibility and planning of a definitive trial to evaluate the effectiveness and cost-effectiveness of the intervention. Lessons learned from this feasibility trial may help others more correctly estimate the timelines and workload involved in setting up and running a multi-centre cluster-randomised controlled trial in the NHS.

## References

[B1] Intercollegiate Stroke Working Party National Sentinel Stroke Audit 2008 2009 London: Royal College of Physicians

